# Monitoring socioeconomic inequity in maternal health indicators in Egypt: 1995-2005

**DOI:** 10.1186/1475-9276-8-38

**Published:** 2009-11-08

**Authors:** Zeinab Khadr

**Affiliations:** 1 Social Research Center, American University in Cairo, Egypt; 2 Department of Statistics, Cairo University, Cairo, Egypt

## Abstract

**Background:**

Egypt's longstanding commitment to safe motherhood and maternal health has paid off in substantial declines in maternal mortality ratio and significant improvement in the levels of many maternal health indicators. The current study aims to monitor trends of maternal health indicators and their socioeconomic inequities among Egyptian women over ten-year period (1995-2005). It poses the question "to what extent have the recent maternal health improvements been shared among the various socioeconomic categories of women?"

**Methods:**

The current paper uses data on maternal health available in three consecutive Demographic and Health Surveys (1995-2000-2005). Concentration index is used to assess the levels of health inequity over the ten year period.

**Results:**

Although previous efforts in maternal health have contributed to substantial improvements in the general levels of maternal health indicators, these improvements were not enjoyed equally by women in various social groups. Indicators that have long been the focus of health policy such as fertility and contraceptive use showed some declines in disparities but they are far behind from achieving equity. Other indicators which relate to unmet need, prenatal care, delivery, postnatal care still loaded with high levels of inequity and call for more comprehensive policy interventions.

## Introduction

Recently, heightened concerns for health equity and social determinants of health have contributed to the burgeoning of evidence-based research that directs policy-makers' attention to the significance of health inequities and promotes policies and actions to tackle their structural and social determinants. In the area of maternal health research, examination of socioeconomic maternal health inequities and disparities has received significant attention among researchers and numerous studies in the international literature have investigated various dimensions of maternal health inequities by women's socioeconomic indicators. These studies have repeatedly confirmed the strong association between women's socioeconomic status and their maternal health indicators, in which low socioeconomic status is significantly associated with low maternal health status and under utilization of maternal health care services [1-4]. In Ecuador, high education and high economic status were positively associated with receiving prenatal care [5]. Women with a secondary level of education or higher were 3.3 times more likely than women with no formal education to receive prenatal care. They were also 3.2 times more likely to receive an adequate number of prenatal visits (The "adequate number of visits" is defined to be 4 times for full gestation time but was also adjusted to the length of gestation). Furthermore, women with high economic status were 26% more likely than low economic status women to receive prenatal care and 61% more likely to receive prenatal care. In Bangladesh, women with more than ten years of education were 2.69 times more likely to use skilled birth attendants (SBA) than women with less than four years of education and those in the highest wealth quintile were almost three times more likely to use SBA than women in the lowest wealth quintile[6]. The same study showed that education and wealth were positively associated with having C-section delivery. Highly educated women were two times more likely to have C-section than uneducated women and women in the highest wealth quintile were 2.64 times more likely to have C-section as compared to women in the lowest wealth quintile. In a study on monitoring maternal and child health Millennium Development Goals in six countries (Cambodia, Dominican Republic, Ethiopia, Ghana, Kenya and Tajikistan), strong associations between education and wealth and various indicators of maternal health were found [7]. Education and wealth quintiles were significantly correlated to usage of SBA and contraceptive prevalence. The same study showed that in Ethiopia, only 3% of uneducated women were using SBA compared to 10% of those with primary education and 45% of women with secondary education. In Kenya, the non-poor women were twice as likely as the poor to have SBA. For contraceptive use, the data revealed a clear educational gradient. However, contraceptive use by wealth quintile revealed a relatively more equitable distribution, although higher prevalence was observed among the richer groups. Furthermore, all countries in their study with information on age at first marriage exhibited a significant educational gradient with women with secondary education marrying at least one year and, in some cases, four years later than women with no education. In reviewing evidence for the need for family planning in several countries, it was concluded that contraceptive prevalence among women in the highest wealth quintiles was almost two to seven times more than its level among women in the lowest wealth quintile [8]. However, these differences were found to become smaller in countries where contraceptives are widely available and accessible. An examination of the relationship between female education and the level of unmet need in South Asia showed that in Bangladesh, India, Nepal and Pakistan, unmet need for family planning is lower among women with secondary and higher education than among women with little or no education [9]. However, the pattern of the relationship between education and unmet need differs by the type of unmet need. In all countries, the unmet need for limiting was more prevalent among women with little or no education, while the unmet need for spacing was more prevalent among women with primary education followed by those with secondary education, except in Pakistan. For instance, in Nepal, the level of unmet need was 31.1% among the uneducated women and was 27.8% among those with secondary education. However, unmet need for limiting was 18.4% among uneducated women and was only 8.8% among those with secondary education and higher, while unmet need for spacing was 12.7% among the uneducated and mounted to 19% among those with secondary education. In Pakistan, both types of unmet need were prevalent among women with little or no education.

With the presence of these large maternal health inequities, many researchers argued that the achievement of Goal 5 of the Millennium Development Goals ("reduce maternal mortality and improve maternal health") can only be materialized in some countries through pro-poor maternal health policies that would enhance vulnerable women groups' access and utilization of maternal health care services [10].

Within the context of Arab countries, disparities in maternal health have been a point of concern among researchers for a long time. These concerns were implicitly elaborated in the conventional tabulation of disparities in maternal health indicators by socioeconomic status in reports of large surveys such as Demographic and Health Surveys (DHS), Pan Arab Project for Child Development (PAPCHILD), Pan Arab Project for Family Health (PAPFAM), and Gulf Family Health Surveys (GFHS), as well as various research studies on maternal health disparities [11,12]. Women's education was found to be the second best predictor of prenatal care and hospital delivery in both Tunisia and Morocco [13]. In Tunisia, women with some education were 40% more likely to receive prenatal care and 46% more likely to deliver in a hospital compared to women with no education, while in Morocco, the effect of women's education was stronger as educated women were 68% more likely to receive prenatal care and 76% more likely to deliver at a hospital than women with no education. In Jordan, high educational attainment and high economic status were significantly related to receiving prenatal care and hospital delivery [14]. Women with high educational attainment and high standard of living were 50% more likely to receive antenatal care and deliver in a hospital than uneducated women and those with low standard of living. In Oman, education was strongly related to women's use of contraceptives [15]. Women with university education were found to be almost four times more likely to use contraceptives compared to uneducated women. Fertility was found to be lower among young Palestinian women (15-24 years) with secondary education or more when compared to other women with less education in Gaza, the West Bank, Jordan and Lebanon [16]. In reviewing hospital-based caesarean sections in the Arab region and Egypt, recent studies unveiled a general positive association between education and Caesarean section [17,18]. Educated women were found more likely to undergo this surgery than less educated women.

More recently, explicit examination of maternal health equities in the Arab countries has only been examined in the international comparative research on maternal health inequities [19]. Using data for 45 developing countries, a recent study showed that Egypt, Morocco and Yemen were among the countries in which significant inequities by wealth quintiles in "births with a professional delivery attendant" and "births for which two or more antenatal visits to a medical professional were received" were found. Clearly, whether discussing disparities or inequities, the literature has generally confirmed the strong association between educational attainment, wealth and maternal health within the context of the Arab countries, consistent with international literature [19].

However, although almost all the earlier literature has succeeded in highlighting the significant disparities/inequities in maternal health indicators, it was mainly concerned with investigating these disparities at one point in time and, in most cases, for small number of maternal health dimensions. In their final report, one of the main recommendations of the WHO Commission on Social Determinants of Health (CSDH) is the need to not only measure health inequities but also to monitor the changes in health inequities over time [20]. The commission further proposed a comprehensive national health equity surveillance framework through which health inequities are monitored over time using five groups of health stratifiers [20].

Along with these recommendations, the current research aims to monitor socioeconomic inequities in maternal health indicators in Egypt. It poses the question "to what extent have the recent improvements in maternal health indicators been shared among the various socioeconomic categories of women in Egypt?"

## The Setting: The Social Context of Maternal Health in Egypt (1995-2005)

Egypt's longstanding commitment to maternal health and safe motherhood has recently paid off in significant declines in the maternal mortality ratio and improvement in the levels of many maternal health indicators. According to Campbell and colleagues and based on two nationally representative maternal mortality surveys, Egypt managed to halve its maternal mortality ratio from 174 per 100,000 live births in 1992-3 to 84 per 100,000 live births in 2000 [21]. The authors attributed this success to "the enormous improvements" in maternal health interventions carried out by the Ministry of Health and Population and the significant improvement in the general social context for women that allowed better utilization of health care services, improved knowledge on maternal health issues as well as better health conditions in general during this period.

More recently, in its midpoint assessment report of Millennium Development Goals (MDGs) 2008, the Egyptian Ministry of Economic Development (2008) indicated that Egypt is almost on the brink of achieving Goal 5 on maternal health of the MDGs [22]. The same report also reveals significant improvements in many maternal health indicators including increases in deliveries attended by skilled birth attendants, deliveries in health facilities, utilization of antenatal care and declines in unmet needs. Nevertheless, the report refers to the presence of disparities in use of maternal health care as one of the major challenges that needs to be addressed. It goes further to postulate that low levels of maternal health care utilization among women with low socioeconomic status are an explicit manifestation of maternal health inequity that requires thorough investigations and monitoring over time.

Improvements in maternal health indicators in Egypt over the ten-year period (1995-2005) came in parallel with significant social policy changes that aim to improve women's life chances as well as creating a more accommodating social context for improving their maternal health status in general. These policy changes were mainly concerned with the empowerment of women through recognizing women's rights in the family; better access to education; involvement of women and young people in the development and implementation of programs and services; and reaching out to the poor, the marginalized and the excluded. They involved active collaboration between both governmental and non-governmental organizations and were manifested in ground-breaking initiatives and reforms. "Girls' Education Initiative" was one of these initiatives. It was adopted by the National Council for Childhood and Motherhood (NCCM), Ministry of Education and UNICEF in 2002 and played a significant role in overcoming cultural and traditional barriers hindering girls' enrolment in school [23]. It was built on three main pillars: namely community mobilization and raising awareness, poverty alleviation, and provision of quality schooling to encourage parents and the community generally to break with custom and tradition, and to think through the implications that girls schooling might have on the household economy and division of labor.

The establishment of the National Council for Women (NCW) in the year 2000 by Presidential Decree No. 90 as an autonomous entity with the purpose of advancing the status of Egyptian women and enhancing their contribution to the growth and development effort of the country represents another significant milestone in the life of Egyptian women. Since its establishment, it has been actively engaged in various activities that extend to numerous dimensions in Egyptian women's lives such as improving their lives socially and economically as well as safeguarding their equal rights. The NCW also collaborated with the Ministry of Health and Population in various activities including upgrading some health facilities and raising health awareness regarding some silent killer diseases [24]. The recent family law reform is another important and significant social development in the social context of the Egyptian families and women in particular. These reforms have been directed to address gaps in existing family and personal status laws that continuously undermined women's right to fair and timely resolutions of family disputes and denied women equal rights within the family [25]. These reforms granted women the right to matrilineal transfer of nationality, the right to file for no-fault divorce and to file for divorce in unregistered marriages. The reforms also simplified and enhanced the effectiveness of the litigation process and the implementation of court orders [25].

In addition, within the same period, the socioeconomic profile of Egyptian women has improved. On the educational attainment front, illiteracy among women aged 15 and older declined 13 percentage points, gross enrolment ratio for basic and secondary education increased from 79% in 1996 to 88% in 2004 and enrolment in secondary education increased from 44.1% in 1996 to 70.1% in 2006 [26,27]. Economically, women's participation in the labor market increased slightly over this period from 22.5% to 23.9% [26,27]. Nevertheless, their occupational structure has exhibited significant changes. According to the Egyptian Human Development Report 2008, women account for one-fourth of the professional and managerial staff in Egypt. Furthermore, the percentage of women working as professionals and technical staff increased from 28% to more than 33% [26,27].

These improvements in the profile of Egyptian women, coupled with the recent social policies changes, are expected to pay off in enhancing the social context for maternal health among Egyptian women. However, even with the presence of a supportive social context for maternal health, it is important to monitor inequities in maternal health in order to provide the policy makers with needed evidence on the impact of the ongoing health and social policies as well as actions aimed at tackling these inequities to identify the main points and areas of interventions. The current study is a modest effort towards monitoring the levels of inequities in the area of maternal health in Egypt.

## Data and Methodology

### Data

The current study uses data from three rounds of the Egyptian Demographic and Health Surveys (1995-2000-2005) to monitor the changes in women's maternal health indicators over the ten-year period (1995-2005). The Egyptian Demographic and Health Surveys are comprehensive reproductive health surveys that aim to provide detailed information on maternal and child health. They have been fielded periodically over the last twenty years in Egypt (1989-1992-1995-2000-2003-2005). The periodicity of these surveys establishes them as a significant and rich data repository for monitoring changes in levels of inequalities in various dimensions of women's reproductive health. The current research is limited to the period of 1995 to 2005 during which Egypt adopted the International Conference on Population and Development (ICPD) plan of action and supportive political commitments and efforts for the implementation of this plan were fully active.

Among the main groups of stratifiers identified by the CSDH recommended national health equity surveillance framework is the socioeconomic group of stratifiers. The commission recommends that it includes education, income/wealth and occupational class. The current study implements educational attainment and wealth as its socioeconomic stratifiers [20]. Limited participation of women in the labor market in Egypt rendered occupational class irrelevant in the current study. Educational attainment is classified into four categories: no education, some primary, primary to secondary and completed secondary and higher. Wealth index is a commonly used measure of economic status of the households in which women live. It is based on the first principle component produced in the factor analysis in a set of indicators regarding the physical characteristics and possession of consumer durable goods for the respondent's household. Wealth index has only been reported in the EDHS (2005). For the purpose of the current study, the wealth index for the EDHS 1995 and EDHS 2000 was calculated using the exact same methodology reported by Macro international for the production of the 2005 wealth index.

Maternal health indicators have been classified into five broad categories of variables; namely early marriage and child bearing, fertility, contraceptive use and unmet need, prenatal care, and delivery and postnatal care.

Analysis in the current study is limited to currently married women who have at least one child or are currently pregnant, which accounts for 86%, 86.5%, and 87% of the total sample in the three surveys, respectively (Table 1). For prenatal and postnatal care, the analysis is restricted to the last birth in the five years prior to the survey.

**Table 1:** Analytical samples for the current study (EDHS 1995-2005)

Year	Total women sample in survey	Married women	Women with living children or currently pregnant	Women with at least birth in the 5 year previous to the survey
1995	14779	13718	12705	7828
2000	15573	14393	13478	7823
2005	19474	18134	16961	9744

### Methodology

The current paper aims to monitor changes in women's maternal health and their levels of inequalities over a ten-year period (1995-2005). Two main measures have been implemented. The first pertains to measuring the women's maternal health in general at three points in time: 1995, 2000 and 2005. For this purpose, simple descriptive measures (means or proportions) are used.

To monitor the changes in the levels of inequality across educational attainment and wealth index, concentration index is used. The concentration index is one of the commonly used measures of inequality in the field of economics. More recently, it became a fairly standard measurement tool in the health economics literature on equity and inequality in health and health care [28]. It allows the measurement of health inequity while taking in consideration the distribution of the health variable across all categories of the health stratifier.

Formally, the concentration index in case of discrete social categories is defined as follows:

where *N *is the number of categories of the social grouping under study, *h*_*i *_is the health variable, *μ *is the mean of the health variable, and *ri *=  is the fractional rank of individual i in the social grouping, with i = 1 for the lowest social grouping and i = N for the highest. For micro level data, a more convenient formula for the concentration index is defined as follows:

where cov is the covariance between the health variable and the fractional rank of the stratifying variable distribution [29]. The concentration index varies between -1 and 1 with zero indicating equality and negative values indicating disproportionate concentration of the health issue among the lowest social stratum compared to the other social strata in the population. In other words, a negative value of the concentration index for "bad" health (ill health) indicates that lower social groupings are more afflicted with this "bad" health. The concentration index usually functions best in the case of a ratio-scale health variable. However, as most of the health variables investigated in the current paper are dichotomous, the boundaries of the concentration index in such a case depend on the mean of the variable [30]. In large samples, it ranges between μ-1 and μ-1, which shrinks the feasible interval for the index. Wagstaff proposed normalizing the concentration index by dividing through by 1 minus the mean to bring back the index boundaries to (-1, 1) [31].

## Results

### Early Marriage and Childbearing

A major concern in maternal health is early marriage and early childbearing behavior. Table 2 shows that the median age at marriage, for currently married women with at least one child in Egypt, was 18 years between 1995 and 2000 and increased by one year in 2005 reaching 19 years. Differentials by educational attainment or wealth index are relatively small (CI = 0.05) and show no changes over the ten-year period, with older age of marriage being more prevalent among the educated and the more affluent women.

**Table 2:** Disparities in age at first marriage and age at first birth, Egypt (1995-2005)

	Year		
	
Indicator	1995	2000	2005
Median age at first marriage	18.0	18.0	19.0
CI by Education	0.05 (0.048 0.052)	0.05 (0.048 0.052)	0.05 (0.048 0.052)
CI by Wealth index	0.05 (0.048 0.052)	0.04 (0.038 0.042)	0.05 (0.048 0.052)
			
% married before age 16***	24.0	20.1	15.4
CI by Education	-0.38 (-0.392 -0.368)	-0.45 (-0.464 -0.436)	-0.47 (-0.484 -0.456)
CI by Wealth index	-0.34 (-0.356 -0.324)	-0.32 (-0.336 -0.304)	-0.39 (-0.408 -0.372)
			
Median age at first birth	20.0	20.0	21.0
CI by Education	0.04 (0.038 0.042)	0.04 (0.038 0.042)	0.04 (0.038 0.042)
CI by Wealth index	0.04 (0.038 0.042)	0.04 (0.038 0.042)	0.04 (0.038 0.042)
			
% had first pregnancy before age 20 ***	35.2	30.8	26.2
CI by Education	-0.37 (-0.382 -0.358)	-0.40 (-0.412 -0.388)	-0.40 (-0.412 -0.388)
CI by Wealth index	-0.34 (-0.352 -0.328)	-0.30 (-0.312 -0.288)	-0.37 (-0.384 -0.356)

CI refers to the concentration index

*** significant at p < 0.001

95% Confidence intervals for concentration index are presented between parentheses

Another important indicator of early marriage is the proportion of currently married women who were married before 16 years of age. Table 2 also shows that this proportion has declined significantly over the ten-year period from 24% in 1995 to 15% in 2005. This decline reflects a decrease in the prevalence of early marriage among younger cohorts. However, disparities by educational attainment and wealth index reveal that early marriage was still much more prevalent among the least educated and poor women and that the gap between these categories and the better off has increased over the ten years.

It is a common knowledge that teenage pregnancy and childbearing can adversely affect the social wellbeing and health of both young mothers and their babies. Furthermore, children and teenage mothers are often weak and at high risk of illness and death. Table 2 reveals the stability of the median age at first birth over the ten-year period with slight disparities by educational attainment and wealth index (CI = 0.04). Nevertheless, although levels of teenage pregnancy showed significant decline over the ten-year period, from 36% in 1995 to 26% in 2005, disparities by educational attainment and wealth index significantly increased as indicated by the significant increase in the concentration indexes for both stratifiers over the ten-year period and particularly between 2000 and 2005.

### Fertility Levels

For the last 40 years, the main common objective of all population policies in Egypt has been to slow the rapid population growth. In response, most of these policies have directed their full attention toward lowering levels of fertility which has paid off in the declining rates of fertility over the period 1995-2005. Table 3 shows that the average number of live births declined from almost 4.0 children in 1995 to 3.27 children in 2005. The success of previous policies is exhibited in the decline in the average number of living children, from 3.3 children in 2000 to 3.0 children in 2005, after experiencing a phase of stalled fertility decline during the period of 1995 to 2000 [32-34]. Furthermore, the comparison between the number of live births and the number of living children reflects the success in lowering child mortality levels, where the difference between the average number of ever born children and average number of living children declined from 0.54 children in 1995 to 0.27 children in 2005. Nevertheless, Table 3 shows that there are relatively large differentials by level of educational attainment and wealth index in both indicators with the uneducated and poor more likely to have more births and more living children. It also indicates that the magnitude of these differentials persisted and has not changed over the ten-year period.

**Table 3:** Disparities in fertility levels, Egypt (1995-2005)

	Year		
	
Indicator	1995	2000	2005
Average number of ever born children***^a^	3.96 (2.8)	3.71 (2.4)	3.27 (2.1)
			
CI by Education	-0.14 (-0.146 -0.134)	-0.15 (-0.154 -0.146)	-0.14 (-0.144 -0.136)
CI by Wealth index	-0.11 (-0.115 -0.104)	-0.09 (-0.096 -0.084)	-0.09 (-0.096 -0.084)
			
Average number of living children***^a^	3.42 (2.0)	3.30 (2.0)	3.00 (1.8)
			
CI by Education	-0.12 (-0.124 -0.116)	-0.13 (-0.136 -0.124)	-0.12 (-0.124 -0.116)
CI by Wealth index	-0.08 (-0.086 -0.074)	-0.07 (-0.076 -0.064)	-0.08 (-0.084 -0.076)

CI refers to the concentration index

*** significant at p < 0.001

95% Confidence intervals for concentration index are presented between parentheses

^a ^Standard error between parentheses

### Contraceptives Use and Unmet Need

A long history of family planning efforts in Egypt has paid off in terms of increases in the levels of ever and current use of contraceptives. Between 1995 and 2005, ever use increased from 73.6% to 84.4% and current use increased from 48.8% to 60.2%. Table 4 also shows that although substantial differentials existed in ever or current use of contraceptives in 1995, these differentials have decreased systematically over the ten-year period. Differentials by educational attainment declined by 14 percentage points for ever use and 7 percentage points for current use of contraceptives. Differentials by wealth index also exhibited significant periods of decline over the ten years. Ever use concentration index showed a decline of 50% between 1995 and 2005, while the concentration index for current use declined by more than 60%.

**Table 4:** Disparities in use of family planning and intention to use among non users, Egypt (1995-2005)

	Year		
	
Indicator	1995	2000	2005
Ever use of modern contraceptives***	73.6	80.4	84.4
CI by Education	0.19 (0.184 0.196)	0.10 (0.096 0.104)	0.06 (0.065 0.064)
CI by Wealth index	0.38 (0.374 0.386)	0.31 (0.306 0.313)	0.19 (0.186 0.194)
			
Current use of modern contraceptives***	48.8	57.3	60.2
CI by Education	0.13 (0.120 0.140)	0.09 (0.082 0.098)	0.07 (0.064 0.076)
CI by Wealth index	0.25 (0.240 0.260)	0.16 (0.152 0.168)	0.10 (0.092 0.108)
			
Average number of children at first use of contraceptives***^a^	2.33 (1.7)	2.28 (1.7)	1.8 (2.3)
CI by Education	-0.17 (-0.176 -0.164)	-0.17 (-0.176 -0.164)	-0.15 (-0.158 -0.142)
CI by Wealth index	-0.16 (-0.168 -0.152)	-0.13 (-0.135 -0.124)	-0.13 (-0.139 -0.122)
			
No intension to use contraceptive among non users***	36.4	34.2	31.8
CI by Education	-0.25 (-0.266 -0.234)	-0.24 (-0.258 -0.222)	-0.23 (-0.250 -0.210)
CI by Wealth index	-0.08 (-0.098 -0.062)	-0.03 (-0.052 -0.008)	-0.03 (-0.052 -0.008)

CI refers to the concentration index

*** significant at p < 0.001

95% Confidence intervals for concentration index are presented between parentheses

^a ^Standard error between parentheses

Increasing levels of contraceptive use were also accompanied by women adopting contraceptive use early in their reproductive lives. Table 4 shows that the average number of children at first use decreased by almost half a child over the ten-year period from 2.3 children in 1995 to 1.8 children in 2005. However, differentials by educational attainment and wealth index maintained their relative magnitudes over the ten years. These differentials indicate that uneducated and poor women are more likely to start using contraceptives after having had more children than educated and more affluent women. Table 4 shows that, for both stratifiers, the concentration index declined slightly over the ten-year period. Less than a three percentage point decline is observed between 2000 and 2005 for differentials by educational attainment and between 1995 and 2000 for differentials by wealth index.

Declines in the proportion of no intention to use contraceptive among non users is considered an indication of the success of the family planning efforts. The proportion of women who never used contraceptives and have no intention to use in the future changed significantly from 36.4% to 31.8% between 1995 and 2005. However, two different patterns of differentials emerge for educational attainment and wealth index. Differentials by educational attainment revealed high prevalence of no intention of contraceptive use in the future among the uneducated women that remains constant over the ten-year period. In contrast, differentials by wealth index were only relative moderate in 1995 (CI = -0.08) that declined by 62% in 2000 and remained constant at 2005.

Unmet need is another criterion for the success of family planning efforts in Egypt. Table 5 shows that levels of unmet need in general and unmet need for limiting and for spacing have declined over the ten-year period by almost one-third. It also shows that although unmet need for spacing is not high (3.8% in 2005), there are increasing differentials by wealth index indicating higher reports of unmet need for spacing among poor women compared to other women.

**Table 5:** Disparities in unmet need and intendedness of last birth, Egypt (1995-2005)

	Year		
	
Indicator	1995	2000	2005
Unmet need for spacing***	6.44	3.64	3.77
CI by Education	0.02 (-0.015 0.055)	0.02 (-0.027 0.067)	0.05 (0.091 0.009)
CI by Wealth index	-0.14 (-0.175 -0.105)	-0.18 (-0.229 -0.131)	-0.17 (-0.209 -0.131)
			
Unmet need for limiting***	11.66	8.16	7.29
CI by Education	-0.20 (-0.224 -0.176)	-0.22 (-0.247 -0.193)	-0.20 (-0.225 -0.175)
CI by Wealth index	-0.24 (-0.265 -0.215)	-0.16 (-0.191 -0.128)	-0.13 (-0.159 -0.101)
			
Unmet need***	18.1	11.8	11.1
CI by Education	-0.13 (-0.150 -0.110)	-0.15 (-0.174 -0.126)	-0.12 (-0.144 -0.096)
CI by Wealth index	-0.23 (-0.250 -0.210)	-0.17 (-0.195 -0.145)	-0.15 (-0.174 -0.126)
			
Unintendedness of last birth***	14.97	9.06	8.24
CI by Education	-0.21 (-0.230 -0.190)	-0.22 (-0.245 -0.195)	-0.17 (-0.195 -0.144)
CI by Wealth index	-0.20 (-0.222 -0.178)	-0.20 (-0.229 -0.171)	-0.18 (-0.207 -0.152)

CI refers to the concentration index

*** significant at p < 0.001

95% Confidence intervals for concentration index are presented between parentheses

Unmet need for limiting is the dominant type of unmet need among Egyptian women comprising almost two-thirds of these needs. Differentials in the unmet need for limiting by women's educational attainment and wealth index are substantial, with a higher prevalence of this type of unmet need among the uneducated and poor women. While educational differentials in unmet need for limiting have maintained their high levels over the ten-year period (CI ≥ 0.20), wealth differentials exhibited a decline over the period with a decrease in CI from 0.24 in. 1995 to 0.13 in. 2005.

Another way to measure the levels of unmet need is the intendedness status of the last birth. Table 5 shows significant declines in the proportion of unintended births from 15% in 1995 to less than 9% in 2005. Differentials by educational attainment and wealth index exhibited small declines over the ten-year period.

### Prenatal Care

Currently married women who had a birth in the five years prior to the surveys were asked about receiving any prenatal care and the content of the prenatal care that they received for their last birth. Table 6 shows substantial increase in the proportion of women who received any prenatal care. The proportion increased from 42% in 1995 to 71% in 2005. However, differentials in receiving prenatal care maintained their high levels over the ten-year period with CI for both stratifiers exceeding 41% in 2005.

**Table 6:** Disparities in prenatal care and its contents, Egypt (1995-2005)

	Year		
	
Indicator	1995	2000	2005
Received any prenatal care ***	42.4	54.1	71.4
CI by Education	0.41 (0.396 0.424)	0.39 (0.378 0.402)	0.41 (0.402 0.418)
CI by Wealth index	0.50 (0.486 0.514)	0.37 (0.358 382)	0.45 (0.442 0.458)
			
Received regular prenatal care (4+ visits)***	31.0	40.7	61.3
CI by Education	0.49 (0.474 0.506)	0.44 (0.426 0.454)	0.39 (0.382 0.398)
CI by Wealth index	0.60 (0.584 0.616)	0.42 (0.404 0.436)	0.49 (0.482 0.498)
			
Received tetanus injection before birth***	73.1	78.0	80.7
CI by Education	-0.15 (-0.162 -0.138)	-0.14 (-0.148 -0.132)	-0.21 (-0.216 -0.204)
CI by Wealth index	-0.22 (-0.234 -0.206)	-0.23 (-0.240 -0.220)	-0.36 (-0.368 -0.352)
			
Told about pregnancy complications***	-	26.0	36.7
CI by Education	-	0.11 (0.083 0.137)	0.12 (0.104 0.136)
CI by Wealth index	-	0.07 (0.041 0.099)	0.15 (0.132 0.168)
			
Given iron supplement during pregnancy***	-	43.6	64.7
CI by Education	-	0.23 (0.212 0.248)	0.17 (0.162 0.178)
CI by Wealth index	-	0.23 (0.212 0.248)	0.22 (0.200 0.240)

CI refers to the concentration index

*** significant at p < 0.001

95% Confidence intervals for concentration index are presented between parentheses

Improvement in receiving prenatal care was also matched with increases in receiving regular prenatal care defined as at least four visits before delivery. The proportion who received regular prenatal care doubled over the ten-year period. Differentials by educational attainment and wealth index, although still very large, declined over the ten-year period with the CI for both having decreased by 10 percentage points.

In these surveys, women are usually asked about the contents of their prenatal care. Table 6 explores three important features of these prenatal care visits. It shows that the proportion of pregnant women who receive tetanus injections has increased over the period, reaching 80% coverage of pregnant women. Differentials by educational attainment or wealth index, although small in magnitude, maintained their levels with higher prevalence of these injections among the uneducated and the poor.

Information on pregnancy complications were more increasingly provided to pregnant women. However, Table 6 shows that this type of information is more likely to be provided to educated and affluent women and the tendency to privilege affluent women with such information is also increasing over time.

Receiving an iron supplement during their pregnancy is another important feature of prenatal care. Table 6 shows that the proportion receiving an iron supplement increased by more than 50% between 2000 and 2005. Although differentials by educational attainment and wealth index were large in 2000, by 2005, only differentials by educational attainment decreased.

### Delivery and Postnatal Care

Over the ten-year period under investigation, deliveries assisted by skilled birth attendants increased by almost 70% from 42% in 1995 to 71% in 2005. This increasing trend was also accompanied by a decline in home delivery from more 65% in 1995 to 34% in 2005. However, for deliveries assisted by skilled birth attendant, although differentials by educational attainment and wealth index exhibit more than a 25% decline, they continue to be very high (CI>0.37) with the more educated and more affluent having deliveries assisted by skilled birth attendant. For home delivery, differentials by educational attainment and wealth index have maintained their high levels over the ten-year period, with the poor and uneducated women more likely to have home deliveries compared to affluent and educated women.

Table 7 also reveals a three-time increase in C-Section delivery over the ten-year period from 7.6% in 1995 to 22.6% in 2005, which poses the question to what extent this increase is due to women's preference, efficiency in detecting risky delivery cases or medical abuse of this procedure [31]? Differentials by educational attainment and wealth index, although showing some declines, maintained their high magnitude (CI = 0.26 by educational attainment and CI = 0.24 by wealth index). These results are consistent with earlier studies that provided evidence that more affluent and educated women were more likely to have C-section deliveries, even controlling for other important factors [6,17,18].

**Table 7:** Disparities in delivery and postnatal care, Egypt (1995-2005)

	Years		
	
Indicator	1995	2000	2005
Skilled birth attendant ***	41.7	55.8	70.5
CI by Education	0.41 (0.396 0.424)	0.38 (0.370 0.390)	0.37 (0.362 0.378)
CI by Wealth index	0.55 (0.536 0.564)	0.42 (0.408 0.432)	0.47 (0.462 0.478)
			
Delivery at home***	64.5	49.1	33.6
CI by Education	-0.42 (-0.430 -0.410)	-0.37 (-0.382 -0.358)	-0.35 (-0.364 -0.336)
CI by Wealth index	-0.53 (-0.540 -0.520)	-0.41(-0.422 -0.398)	-0.47 (-0.484 -0.456)
			
Who had C-section delivery ***	7.6	11.4	21.6
CI by Education	0.31(0.271 0.349)	0.27(0.237 0.303)	0.26 (0.227 0.293)
CI by Wealth index	0.40 (0.361 0.439)	0.29 (0.253 0.327)	0.34 (0.144 0.536)
			
Checked by a physician after delivery***^1^		58.6	72.7
CI by Education		0.39 (0.380 0.400)	0.36 (0.354 0.366)
CI by Wealth index		0.41 (0.400 0.420)	0.48 (0.472 0.488)
			
Received vitamin A supplement after delivery ***	-	12.1	48.8
CI by Education	-	0.20 (0.169 0.231)	0.07 (0.058 0.081)
CI by Wealth index	-	0.18 (0.145 0.215)	0.04 (0.028 0.052)

CI refers to the concentration index

1 This proportion includes both women who were assisted by a doctor during delivery or were checked by a doctor after delivery

*** significant at p < 0.001

95% Confidence intervals for concentration index are presented between parentheses

Two features of postnatal care were investigated: namely being checked by a doctor after delivery and receiving a vitamin A supplement after delivery. Table 7 shows that the occurrence of a medical check by a doctor after delivery increased 25 percentage points between 2000 and 2005 which might be attributed to the increase in the prevalence of deliveries assisted by skilled birth attendant. Differentials by both stratifiers maintained their high levels favoring the more educated and affluent women. Receiving vitamin A supplement shows a substantial improvement both on the average level and on the differentials by educational attainment and wealth index. Over a period of five years, the proportion of women who received vitamin A supplement increased four times from 12% in 2000 to 49% in 2005. Differentials by educational attainment and wealth index also declined by more than 75% and 85%, respectively, approaching more equitable status.

## Discussion

There is no doubt that safe motherhood and maternal health are reproductive rights that ought to be enjoyed by all women in their reproductive age and even beyond. The current study proved that within a policy supportive context, efforts to improve maternal health in Egypt paid off in terms of improved general levels of almost all maternal health indicators. Nevertheless, the current study also provided sufficient evidence that these general improvements in maternal health were not equally enjoyed by all population groups and highlighted substantial inequities among women by educational attainment and wealth in the majority of maternal health indicators examined. Similar to findings from earlier studies in the international literature [1-10], uneducated and poor women were more likely to marry and have children at young age, have more children and were less likely to use contraceptives, prenatal care and postnatal care services than educated and affluent women. The study also confirmed the positive relationship between women socioeconomic status and having a C-section reported in earlier studies [6,17,18].

Additionally, the study revealed that the inequities and their trends vary by the dimension of maternal health investigated. Age related dimensions of maternal health proved that averages can conceal large inequities. While both the median age at first marriage and the median age at first pregnancy, exhibited small and stable inequities, inequities were large and increasing when the investigation focused on these incidences for a specific age brackets. For the levels of fertility, contraceptive use and unmet need, the inequities by both stratifiers were moderately high and to a large extent stable over the period. Use of prenatal care revealed substantially stable or slightly declining inequities with the concentration index for its indicators ranging between 0.41 and 0.60 in 1995 and between 0.39 and 0.49 in 2005. Regarding the content of prenatal care, the study revealed that poor and uneducated women were increasingly more likely to receive tetanus injection, while more affluent and educated women were more likely to be informed about pregnancy complications and receive iron supplements. Inequities in delivery and postnatal care were substantially high with the range of the concentration index extending between 0.26 and 0.48 in 2005. The study also revealed an important finding regarding Caesarean delivery. It showed that while the prevalence of the C-section delivery increased over the ten years (from 7.6% in 1995 to 21.6% in 2005), inequities in having this surgery decline by more than 15% by both stratifiers. This rising trend combined with the declining inequities by both stratifiers and the fact that the proportion of Caesarean delivery in 2005 exceeds the World Health Organization "threshold" of 15% clearly raise many questions on the validity of the medical justification for this high prevalence of this surgery. Recent studies showed that the odds of C-section delivery are higher by 12% in private hospitals compared to public hospitals which again raise more questions regarding profitability and convenience of delivery in carrying out this surgery [17,35]. These questions clearly point to the need for further comprehensive studies that aim to investigate this rising trend and assess its underlying reasons.

The study also revealed that although the inequities observed by both stratifiers were closely similar, one can detect certain patterns for each stratifier. Differentials by educational attainment were slightly higher than differentials by wealth for indicators related to marriage, fertility and initiation of and intention for use of contraceptives, while differentials by wealth were higher than those by educational attainment for use of contraceptive, unmet need, access and utilization of prenatal care, delivery and postnatal care. These two patterns need to be taken in consideration in formulating policies and interventions. The first pattern requires more effort towards changing the attitude of uneducated women and attempting to reach them through providing them with the needed information, while the second would imply making maternal services more accessible to poor women either through providing information or cost reduction.

Although the current paper attempted to provide a full picture of the maternal health in Egypt, certain limitations in the current study deserve mentioning. Limitations in the current study come as a result of the restricted focus of the DHS surveys on specific aspects of adult women in reproductive age. These surveys lack discussions of important maternal health issues such as preconception care, pregnancy wastage, and maternal mental health aspect particularly postpartum depression. Furthermore, the fact these surveys are mainly focused on adult women in reproductive age discard other important reproductive stages in women's life particularly during menopausal age and older. In addition, some important indicators of women's maternal health have only been investigated in last two surveys which limit the discussion of trends over the ten-year period.

Finally, the current study and other studies with similar focus with in the context of the Arab countries represent a needed effort to reveal the importance of monitoring the maternal health inequity by simple health stratifiers. These studies clearly highlight the need for more in depth studies that use various health stratifiers, investigate more health dimensions and explore the impact of the various structural and social determinants with the aim of highlighting policy entry points to tackle health inequities.

## Conclusion

Egypt, as one of the earliest Arab countries to adopt population and family planning policies, has managed to achieve significant improvements in almost all maternal health indicators. Evidence for these improvements is clearly observed in the magnitude of these indicators over the ten-year period 1995 to 2005. Unfortunately, these improvements were not matched with diminishing levels of inequity among the socioeconomic groups of women. The current analysis provided clear insights pertaining to the main equity-oriented policy implications for maternal health in Egypt. Future policies need to revise current policies towards more equity-oriented and sensitive policies that take in consideration both the social context in which these women live and factors related to health services. These policies should call upon the engagement of different players including governmental and non-governmental organizations in tackling issues of inequity at the root causes and work out obstacles that hinder women's access and use of maternal health services. Furthermore, with a revival of the commitment to improving primary health care in the international arena, there is a need to reorient the health care system towards providing equitable access to care and efficiency in service delivery as well as achieving the balance between curative and preventive health care. Finally, research that monitors health inequities by various stratifiers should be considered an essential tool in setting future policies in order to allow policy makers to track inequities and identify vulnerable groups and evaluate the impact of the various health and social policies and interventions in achieving health equity.

## Competing interests

The author declares that she has no competing interests.
